# Cryoablation Combined with Left Atrial Appendage Closure: A Safe and Effective Procedure for Paroxysmal Atrial Fibrillation Patients

**DOI:** 10.1155/2020/6573296

**Published:** 2020-04-10

**Authors:** Zhongyuan Ren, Jun Zhang, Mengyun Zhu, Dongdong Zhao, Shuang Li, Haotian Yang, Yixing Zheng, Weilun Meng, Jingying Zhang, Yawei Xu

**Affiliations:** ^1^Department of Cardiology, Shanghai Tenth People's Hospital, Tongji University School of Medicine, Shanghai, China; ^2^Soochow University Medical College, No. 199 Ren-ai Road, SIP, Suzhou, China

## Abstract

**Background:**

Catheter ablation combined with left atrial appendage closure (LAAC) was reported as a feasible strategy for atrial fibrillation (AF) patients with high risk of stroke or contraindications of oral anticoagulants. We aimed to observe the short-term safety and efficacy of combining cryoballoon ablation (CBA) with LAAC in paroxysmal (PAF) patients. *Method and Results*. From Jan 2016 to Dec 2017, 304 patients diagnosed with nonvalvular, drug-refractory PAF were included, who underwent either CBA alone (*n* = 262) or combined procedure (*n* = 42). Instant pulmonary vein isolation (PVI) with CBA was achieved in all patients, while successful LAAC achieved in 41 (97.6%) of combined procedure patients. 1-year freedom of AF rate was lower in combined procedure group (84.7% vs 70.7%, *p* = 0.04), with unadjusted hazard ratio (HR = 1.97) and 95% confidence interval (CI) 1.03–3.77. However, the multivariate COX model revealed left atrial diameter (*p* = 0.002, HR = 1.10, and 95% CI 1.04, 1.17), rather than procedure type (*p* = 0.51, HR = 1.34, and 95% CI 0.57, 3.17), was the predictor for freedom of AF. Only 2 patients in the CBA group had stroke, contributing to the nonsignificant higher stroke incidence (*p* = 1.00). Transoesophageal echochardiography (TEE) achieved in 35 patients (83.3%) showed complete occlusion with no obvious residual flow (>3 mm), Device-related thrombosis, or pericardial perfusion. All-cause mortality, rehospitalization, and complication rates were similar.

**Conclusion:**

Combining CBA with LAAC in a single procedure is a feasible strategy for PAF patients, with comparable short-term safety and efficacy to CBA alone.

## 1. Introduction

Atrial fibrillation (AF) is a commonly faced cardiac arrhythmia, which has been a heavy worldwide burden with a prevalence over 10 million in China [1]. Catheter ablation (CA) was established as the standardized technique to achieve rhythm control for AF patients and recently proven to be noninferior to medications by the CABANA study [2]. Furthermore, since the FIRE AND ICE trial provided the evidence of noninferiority of cryoballoon ablation (CBA) to radiofrequency ablation [3], CBA became a pervasive strategy for CA owning to its smooth learning curve and less center experience dependence [4].

To prevent stroke and embolic events in AF patients with high thrombotic risk (CHA_2_DS_2_-VASc score ≥2 in male and ≥3 in female), oral anticoagulants (OACs) has been suggested to be effective, with class I, level A recommendation [5]. For Chinese population, however, insufficient anticoagulation was the major obstacle of AF management, resulted from the low compliance at a great extent [6]. Furthermore, labile international normalized ratio (INR) in patients taking warfarin and insufficient dosage of new OACs rendered anticoagulation far from enough. In addition, contraindications to long-term OACs and severe adverse effects as major hemorrhagic events limited the application in specific patients. Since left atrial appendage harbors 90% of the thrombus in nonvalvular AF patients [7], left atrial appendage closure (LAAC) is developed as an alternative to achieve stroke prevention. Recently, Dr. Lucas Boersma presented promising 2-year follow-up from the EWOLUTION trial that AF patients with contraindications of OACs benefit significantly from LAAC, reducing both ischemic and hemorrhagic events. LAAC was gradually adopted as a procedure providing effective stroke prophylaxis in nonvalvular AF patients.

Sharing the same venous access of procedure, combining CA with LAAC in a single procedure was firstly reported by Swaans and colleagues in 2012, conducted in 30 AF patients [8]. Thereafter, the feasibility, safety, and efficacy of combined procedure have been consecutively investigated by clinicians. Followed by Alexander Romanov and colleagues, their random controlled trial further proved the efficacy [9]. Combined procedure became a feasible strategy in patients with symptomatic drug-refractory AF, high risk of stroke, or contraindications to long-term OACs [10]. Nevertheless, according to our knowledge, few studies explored the combined procedure in paroxysmal AF (PAF) patients, or specifying cryoballoon ablation strategy. Moreover, no comparison between combined procedure and CBA has been reported.

In the present study, we shared our experience of CBA-combining procedure in PAF patients and validated the safety and efficacy by comparing CBA-combined procedure with CBA only.

## 2. Methods

### 2.1. Studied Population

We retrospectively investigated 399 continuous patients with documented nonvalvular, atrial fibrillation from Jan 2016 to Dec 2017, who underwent either CBA or combined procedure of CBA and LAA closure at Atrial Fibrillation Center of Shanghai Tenth people's hospital. Among them, 323 were diagnosed as paroxysmal atrial fibrillation (PAF) and included. PAF was defined as atrial fibrillation recorded and confirmed either by a 7-day or 24-hour ECG monitor, which converted to sinus rhythm spontaneously or by intervention (cardioversion or antiarrhythmic drugs) within 7 days [5]. Further, we excluded 19 patients who lost to follow-up, and eventually 304 patients were included. Before the procedure, CHA_2_DS_2_-VASc and HAS-BLED scores were calculated for every patient to assess the stroke and hemorrhagic risks. Two-dimensional transthoracic echocardiography (TTE) was conducted to evaluate cardiac function quantitatively, including left ventricular ejection fraction (LVEF), left atrial and ventricular size, and valvular function. Meanwhile, transoesophageal echocardiography (TEE) was applied to evaluate left atrial (LA) size and left atrial appendage (LAA) size and shape, and rule out thrombus in LA or LAA beforehand. Patients considered eligible for combined procedure are as follows: (a) drug-refractory nonvalvular paroxysmal atrial fibrillation patients and (b) patients with one of the following conditions: (a) CHA_2_DS_2_-VASc score ≥2, (b) HAS-BLED score ≥3, (c) contraindications to long-term OACs, and (d) refuse OACs as antithrombotic regimen according to personal willingness. Patients with the following conditions were excluded from interventional procedure: (a) thrombus in LA or LAA presented and confirmed by TEE, (b) oversized LA (LA diameter >65 mm) by TTE or LAA (LAA opening >35 mm) through TEE, (c) pericardial perfusion (volume ≥4 mm by TTE or TEE), (d) hemodynamic unstable patients, (e) patients with active hemorrhagic diseases, and (f) ischemic or hemorrhagic stroke within 30 days. Our study complied the Declaration of Helsinki (1964). From each patient, informed consent was obtained before the procedure, with procedural risks and possible complications fully informed. The study was approved by the Ethical Committee of Shanghai Tenth People's Hospital. Both CBA and LAAC combined procedures were performed by the same proficient electrophysiologist, with CBA procedure over 100 cases and LAA closure 50 cases per year.

### 2.2. Procedure Details

#### 2.2.1. Cryoablation

The procedures were accomplished under local anesthesia by subcutaneous administration of lidocaine at the groin region. Through femoral vein access, guidewire and vessel dilator were applied ensuing a single transseptal puncture. A 23 mm or 28 mm second-generation cryoballoon (Arctic Front, Medtronic, MN, USA) was advanced through a steerable sheath (FlexCath, Medtronic, MN, USA) into the left atrium (LA). Once a pulmonary vein (PV) confirmed, the cryoballoon was inflated and advanced to the ostium of the PV, following angiography by injection contrast dye ensuring complete PV occlusion. Accompanying Achieve catheter (Achieve, Medtronic, Minneapolis, MN) cannulated distally into the PV to detect electric activity and confirm PV isolation (PVI), a standard 180 s freeze was adopted for each pulmonary vein. Freezing time was adjusted according to time to isolation (TTI) recorded. When TTI ≤30 s, freezing time was set to 150–180 s. While TTI between 30 and 60 s, freezing time was set to 180 s. A bonus freeze of 120 s was applied only when TTI >60 s. If TTI could not be recorded, a 180 s freeze would be adopted, with a bonus freeze of 120 s when temperature declined over −40°C before 60 s from application.

Continuous phrenic pacing (8–10 V, 30 times per minute) with electrode placed at superior vena cava was applied during freezing of right superior PV (RSPV) and right inferior PV (RIPV). Through observation of the decrement of diaphragm movement under fluoroscopy, phrenic nerve palsy was detected, when freezing procedure was subsequently halted to prevent further injury. The vital signs were continuously monitored during the procedure. Freezing was instantly halted for esophageal protection, once the patient complained about nausea, vomiting, chest distress, or pain. Additionally, a proton pump inhibitor (PPI) was prescribed to all patients till the 2^nd^ month since the procedure. Heparin was intravenously infused during the whole procedure with monitoring activated clotting time (ACT) between 250 and 350 s.

#### 2.2.2. LAA Closure

**Figure 1:**
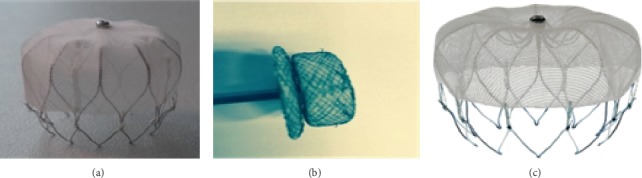
Three types of LAAC devices. (a) Lefort (Shape Memory Alloy Co, Shanghai, China), (b) Lacbes (Shanghai Push Medical Device Technology Co., LTD, Shanghai, China), and (c) Watchman device (Watchman, Boston Scientific, MA, USA).

**Figure 2:**
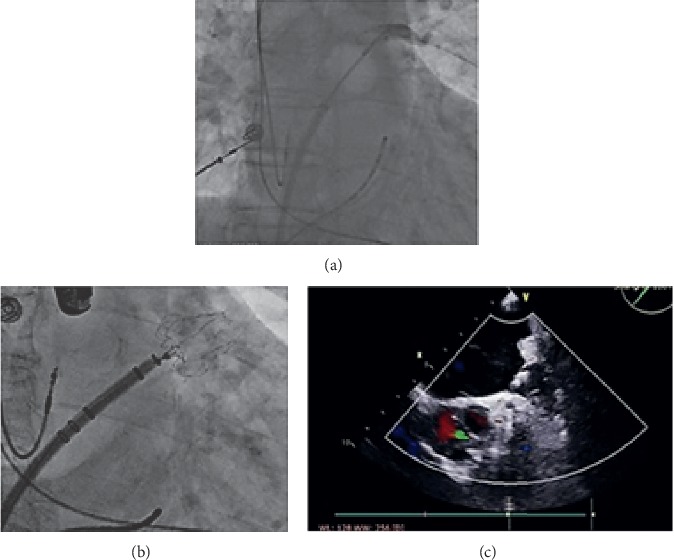
Procedure details about CBA and LAAC. (a) Balloon ablation of left superior pulmonary vein (LSPV) that fully occluded by angiography. (b) Deployment of the WATCHMAN device under fluoroscopy. (c) Transoesophageal echocardiography (TEE) showed complete occlusion without malposition or residual flow.

Under local anesthetic state, an LAA closure device was implanted instantly after CBA in eligible patients. The first 23 patients were implanted with Lefort (Shape Memory Alloy Co., Shanghai, China), followed 11 patients with Lacbes (Shanghai Push Medical Device Technology Co., LTD, Shanghai, China), and latest 8 patients with WATCHMAN (Watchman, Boston Scientific, MA, USA), presented in Figure 1. Generally, followed by the femoral vein access from CBA procedure, 14-F guide wire and pigtail catheters were advanced into the LAA. The size, depth, and shape of LAA was observed and recorded by angiogram under fluoroscopy guidance (RCA 30° + CAU 20°/CRAN 20°) and transesophageal echocardiography (TEE) with angle approximately 0°/45°/90°/135°. According to the measured feature of LAA, the device 10–20% oversize to the LAA was chosen to ensure stable positioning and proper compression. The pigtail catheter was removed after the WATCHMAN access system advanced over into the LAA. Subsequently, the device was carefully delivered in the LAA and deployed at the ostium of the LAA by retracting the access sheath. Before release, PASS principle was fulfilled and confirmed by TEE for all devices, encompassing device position at LAA ostium, stable anchoring confirmed by tug test, and device compressed for 10–25% of the original size, sealed with residual flow ≤3 mm. Once released, LA angiography and TEE recheck for positioning, compression ratio, and residual flow were followed, simultaneously assessing for possible pericardial perfusion.  Figure 2 shows procedure details about CBA and LAAC.

### 2.3. Follow-Up and Postprocedural Management

All patients were informed and continuously followed to reexamine at 1^st^, 3^rd^, 6^th^, and 12^th^ month after the procedure. Meanwhile, transtelephonic follow-up was carried out at the same time point to evaluate individual condition and guarantee timely reexamination. For reexamination at inpatient or outpatient, medical history and physical examination were achieved, and either a 24-hour or 7-day electrocardiograph (ECG) monitor was applied to detect the recurrence of AF and other arrhythmias.

In our study, primary endpoints were stipulated as AF recurrence and stroke incidence, while secondary endpoints included all-cause mortality, acute myocardial infarction, heart failure, pericardial effusion, and device-related thrombosis. Recurrence of AF was defined as AF lasting longer than 30 s, while the time before the 3^rd^ month from the procedure was defined as blank period, when no recorded AF was considered recurrence.

For patients who underwent combined procedure, TEE was performed at the 3^rd^ month and the 12^th^ month to evaluate the positioning of the device, sealing of LAA, device-related thrombosis, and other structual changes.

For postprocedural medications, antiarrhythmic drugs (sotalol) were generally prescribed to every patient during the blank period. Anticoagulation therapy for 2–3 months was recommended to all PAF patients underwent either procedure. For patients underwent combined procedure, warfarin or new oral anticoagulants (NOACs, dabigatran, or rivaroxaban) were prescribed and discontinued for 2–3 months. Once satisfied device positioning, no obvious residual flow (>3 mm) and no thrombus presented were confirmed by TEE; antiplatelet therapy was thenceforth substituted by double antiplatelet treatment (aspirin and clopidogrel) till the 6^th^ month, following single antiplatelet medication as a long-term therapy (aspirin or clopidogrel), while the continuation of OACs in the CBA group was based on the CHA_2_DS_2_-VASc score individually. If CHA_2_DS_2_-VASc ≥2 in male or ≥3 in female, OACs were recommended for long-term anticoagulation.

### 2.4. Statistical Analysis

Continuous variables were described as mean ± standard deviation (SD) or median (interquartile range), and *p* value was analyzed from a two-sample *t*-test if the variance was equal or Mann–Whitney test if not. Categorical variables were described as percentage (%), with *p* value analyzed from the *χ*^2^ test or Fisher exact test if there was theoretical frequency lower than 5. The Kaplan–Meier estimate analyzed the freedom of atrial arrhythmia with *p* value achieved by the log-rank test. Hazard ratio was calculated through the univariate COX proportional hazard regression model. The multivariate COX model (forward and backward stepwise selection with *p* < 0.05) was applied to determine the prognostic factors of the freedom of AF. 2-side *p* value <0.05 was considered significant in all analyses. We used SAS 9.4 software (SAS Institute Inc., Cary, NC, USA) to conduct the analysis.

## 3. Results

We included 42 and 262 patients underwent CBA + LAAC procedure and CBA only, respectively. Baseline characteristics are described in Table 1. Both the CHA_2_DS_2_-VASc score (3.8 ± 2.1 vs 2.8 ± 1.9, *p* < 0.0001) and HAS-BLED score (3.8 ± 2.1 vs 2.8 ± 1.9, *p* < 0.0001) were significantly higher in the combined procedure group than in the CBA only group. Besides, the combined procedure group was elder (70 ± 7.6 yrs vs 66.3 ± 9.5 yrs, *p* = 0.01), with larger LA diameter (45.6 ± 5.8 mm vs 40.4 ± 5.6 mm, *p* < 0.0001), higher prevalence of hyperlipidemia (14.3% vs 3.8%, *p* = 0.01), and previous stroke history (61.9% vs 23.7%, *p* < 0.0001). Routine medications also differed, with higher rate in the combined procedure group, including antiplatelet agents (46.3% vs 27.1%, *p* = 0.01) and statins (31.7% vs 15.6%, *p* = 0.01). Other characteristics were comparable between two groups.

### 3.1. Periprocedure Details

Details about CBA are listed in Table 2. Procedural characteristics including nadir and procedure time were comparable between the two groups. Meantime, it took similar ablation time to achieve PVI between both the groups (16.2 ± 5.1 min for CBA vs 17.7 ± 7.7 min for combined procedure, *p* = 0.47) and similar number of applications. Touch-up ablation of other arrhythmogenic sites was applied in 5 patients in the CBA only group, encompassing 2 at LAA, 1 at superior vena cava, 1 at mitral isthmus, and 1 at tricuspid isthmus.

For periprocedure complications, no obvious pericardial effusion (>3 mm) was observed in both the groups. Vagal reflex monitored by blood pressure and heart rate was observed in 11 (26.7%) and 29 (11.5%) patients and not statistically significant (*p*=0.20). Transient phrenic nerve palsy occurred only in 3 patients in the CBA only group, observed as transient decrease of diaphragm movement in 2 patients and halted diaphragm movement in 1 patient during ablation of the right superior pulmonary vein (RSPV).

Totally 42 patients underwent LAAC immediately after CBA.  Table 3 provides the details of LAAC procedure. The first 23 patients were implanted with Lacbes, followed 11 patients with Lefort, and latest 8 patients with the WATCHMAN device. Overall implantation success rate was 97.6%, where 1 patient failed to implant with the WATCHMAN device due to mismatch between oversized LAA ostium by angiography and suitable device. Device replacement occurred in 2 patients. One patient (LAA opening diameter 21 mm) firstly implanted with a 24 mm Lefort device was observed with obvious residual flow (>3 mm) and subsequently replaced with 27 mm one which deployed and fixed stably without residual flow. A 22 mm Lacbes device was implanted in the other patient (LAA opening diameter 16 mm), which was over compressed (compression ratio >25%). Proper compression ratio was then achieved by replacing a 20 mm device (compression ratio = 20%). 5 patients were observed with minimal residual flow (≤3 mm) after release (3 with Lefort and 2 with Lacbes), confirmed by TEE. Two patients recorded AF during the procedure underwent cardioversion, who were converted to sinus rhythm. No pericardial effusion or other complication was observed.

### 3.2. Follow-Up

**Figure 3:**
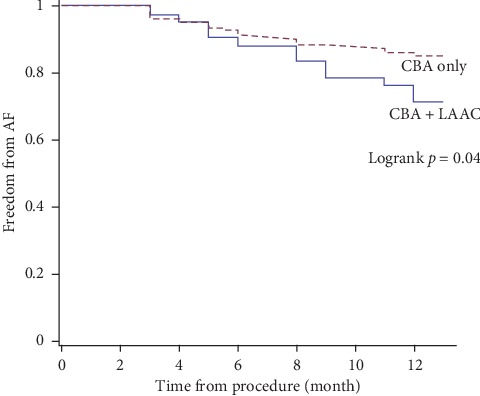
1-year follow-up of freedom of AF.

**Figure 4:**
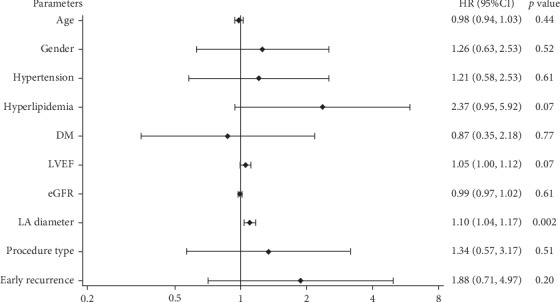
The multivariate COX regression model was done incorporating age, gender, hypertension, hyperlipidemia, DM, LVEF, eGFR, LA diameter, early recurrence of AF, and procedure type (0 for CBA only and 1 for combined procedure). DM, diabetes mellitus; LVEF, left ventricular ejection fraction; eGFR, estimated glomerular filtration rate; LA diameter, left atrial diameter.

Follow-up results are presented in Table 4. Follow-up time ranged from 3 months to 35 months. The median follow-up time was 22 ± 11 months in the CBA only group and 20 ± 9 months in the combined procedure group. Recurrent AF during blanking period occurred in 16 (6.2%) CBA patients and 5 (12.0%) combined procedure patients with *p*=0.30. Correspondingly, 40 (15.3%) and 12 (29.3%) patients experienced late recurrent AF. Among which, 5 patients in the CBA group and 1 patient in the combined procedure group underwent redo-ablation. Survival analysis as presented in Figure 3 showed that 1-year freedom of AF was significantly different between the groups (*p*=0.04, hazard ratio (HR)  = 1.9, 95% confidence interval (CI) 1.0, 3.7). However, the multivariate COX regression model incorporating totally 10 parameters showed only LA diameter (*p*=0.002, HR = 1.10, and 95% CI 1.04, 1.17), rather than procedure type (*p*=0.51, HR = 1.34, and 95% CI 0.57, 3.17), was the significant predictor of freedom of AF (Figure 4).

Among patients underwent LAA closure, 35 (83.3%) patients were reexamined by TEE, showing LAA closure device was well positioned and sealed in all 35 patients. No obvious residual flow nor device-related thrombus was observed. No obvious pericardial perfusion was detected.

Two patients in the CBA only group died at the 3^rd^ month of follow-up, one resulted from intracerebral hemorrhage and the other from interstitial pneumonia. Besides, the rehospitalization (due to cardiovascular events) rate was comparable betweenthe CBA only and combined procedure groups (7.3% vs 4.8%, *p* = 0.79). Notably, although not statistically significant, 2 patients had intracerebral hemorrhage (1 as described above and 1 occurred at the 2^nd^ month from the procedure) and 2 had myocardial infarction (one in 8^th^ month and the other one in 12^th^ month, and percutaneous coronary intervention was carried out in both patients) in the CBA only group. No stroke, myocardial infarction, or systemic embolic event occurred in the combined procedure group.

## 4. Discussion

Combined procedure of CA and LAA closure was developed as a novel and efficient approach which can be manipulated from the same femoral vein access and atrial septal puncture in a single procedure. Meanwhile, cryoablation, as a noninferior [3] and less experience-depended [4] strategy to radiofrequency ablation, was becoming widely adopted to achieve PVI. Based on the evidences, in 2016, the first result of combining CBA with LAAC procedure was published by Gaetano Fassini and his colleagues [11]. Incorporating 35 patients with a follow-up of 24 + 12 months, the recurrence of atrial arrhythmia rate was 28% while no device-related complications or clinical thrombotic event occurred. This was a promising result of combined procedures, nevertheless, demanded further supports from larger scale studies and randomized controlled trials. In our study, we provided our experience and validated the efficacy and safety of combined procedure.

### 4.1. Studied Population

Our study incorporated 304 nonvalvular PAF patients underwent either CBA only or combined procedure at our center. Because the patient was considered eligible for combined procedure only when CHA_2_DS_2_-VASc ≥3, or HAS-BLED score ≥2, or strictly or relatively contraindicated OACs, there were significant differences at baseline in combined procedure group compared with CBA only group, including advanced age, high CHA_2_DS_2_-VASc and HAS-BLED scores, large LA diameter, high prevalence of previous stroke, and high proportion of anticoagulants intake. In addition, the prevalence of hyperlipidemiaand the proportion of statins intake were higher in the combined procedure group as well, in line with the inverse relation between dyslipidemia and AF incidence during follow-up [12].

### 4.2. Periprocedure Evaluation

**Figure 5:**
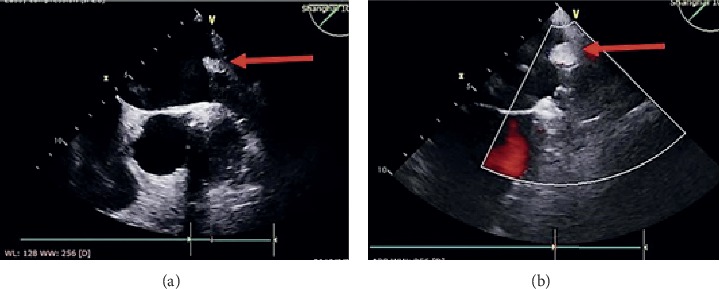
Pulmonary vein-left atrial appendage ridge before and after CBA by TEE. Distinct ridge mass was observed comparing TEE evaluation (a) before (red arrow) and (b) after CBA (red arrow).

Successful PVI by CBA was achieved in all patients, and LAAC achieved in 97.6%, with 1 patient failed to implant due to unmatched device size with LAA. The success rate was in accordance with studies reported [9, 11]. Only transient phrenic nerve injury occurred in 3 patients of the CBA only group and postprocedure electroversion applied in 2 patients in the combined procedure group after LAAC who converted to sinus rhythm, while there was no significant difference of periprocedure complication rate between the groups. Additionally, 5 patients had minimal residual flow (≤3 mm) detected by TEE after implantation, which was defined as flow ≤5 mm in PREVAIL and PROTECT AF trials and proven to have no influence on placementof the device or thrombosis [13, 14]. Interestingly, distinct pulmonary vein ridge edema was observed after CBA procedure by TEE (Figure 5), even though deployment and release of WATCHMAN, Lefort, and Lacbes were unacted.We believe such phenomenon could interfere the implantation of LAAC devices that require overlaying LAA opening, such as Amplatzer Cardiac Plug (ACP) or LAmbre. Furthermore, we predicted that the incidence of device displacement and obvious residual flow would increase owning to the subsiding of edema, although not observed by TEE at the 3^rd^ month follow-up since the procedure. In spite of the results, we still recommend plugging a LAAC device such as WATCHMAN as a first choice when considering LAAC immediately after cryoablation, in order to avoid device displacement after the ridge edema subsided. Long-term evalutation is warranted to provide stronger evidences. 

### 4.3. Safety and Efficacy

With a meanfollow-up time of 22 ± 11 months in the CBA only group and 20 ± 9 months in the combined procedure group, the recurrence of AF was significantly higher in the latter (15.3% vs 29.3%, *p* = 0.04) and procedure type was indicated as a predictor of freedom of AF as well (HR = 1.9, 95% CI 1.0, 3.7). As discussed above, the baselinecharacteristics differed, including CHA_2_DS_2_-VASc [15] scores, LA diameter [16], and the prevalence of hyperlipidemia, which were significantly related to AF recurrence in a univariate model. However, through adjusting confounding parameters, the multivariate COX model showed only LA diameter (*p* = 0.002, HR = 1.10, and 95% CI 1.04, 1.17) rather than procedure type (*p* = 0.51, HR = 1.34, and 95% CI 0.57, 3.17), was the predictor of freedom of AF. Our results showed combining LAAC in CBA procedure could achieve AF rhythm control with comparable efficacy to CBA alone. . More trials are warranted to support the conclusion.

Considering stroke prophylaxis, our results showed that combined procedure seemed to be effective with a nonsignificant lower stroke risk (only 2 (0.7%) in the CBA only group). Of note, both cases were resulted from lethal intracranial hemorrhage. Results from the CABANA trial showed LAAC performed effectively in preventing hemorrhagic stroke [17]. Although we observed similar tendency in our study, prevention of hemorrhagic stroke needs to be validated in larger, prospective studies with longer follow-up period.

Among 35 patients underwent LAAC assessed by TEE, no device-related thrombosis, obvious residual flow, or complications as pericardial perfusion was observed. Besides, severe vascular events were only observed in the CBA only group, where 2 patients who had intracerebral hemorrhage and 2 who had myocardial infarction underwent PCI. The low incidence rate of complication of combined procedure was comparable to studies with or without specifying the ablation strategy [13, 14, 18]. Hence, we believe combined procedure would not bring addtional complcations to LAAC. 

From the aspect of anticoagulation, all patients received OACs therapy were discontinued (either warfarinor NOACs) for at least 2 months since the procedure in the combined procedure group, whether or not shifted to antiplatelet therapy after the reexamination by TEE at the 3^rd^ month. Although single LAAC procedure was recommended with anticoagulation (either warfarinor NOACs) for at least 45 days [19], as there was no available guideline to recommend the proper dose and duration for patients underwent combined procedure, it was prolonged to 2 months for the patients and proven to be safe with adequate stroke prevention in most trials [11, 20]. Moreover, recently, a case reported by Steven K. Carlson presented that 45-day anticoagulation was not adequate to prevent thrombosis in patients combined procedure [21]. Hence, based on our study, in nonvalvular PAF patients combining CBA with LAAC, the anticoagulation regimen of at least 2 months either by VKA or NOACs and substituted by double antiplatelet therapy and then lifelong single antiplatelet therapy was a considerable choice for prevention of thrombotic events, without increasing risk of hemorrhagic events.

## 5. Limitations

Our nonrandomized, retrospective study design limited the application of the conclusion. With relatively small number of combined procedure patientsand specific inclusion criteria, the conclusion cannot be extrapolated in gerenal AF population. Meanwhile the incidence of complications like stroke might be underestimated due to short follow-up period.Additionaly,TEE was available only in 35 (83.3%) of combining procedure group, the device-related complication rates might be higher than reported. Owning to the intolerance of TEE, a better evaluation approach and follow-up strategy should be considered in order to ensure safety and relief patients' painfulness.Furthermore, we applied 3 types of LAAC devices in our study, with only 1 case failed to implant. While the safety and efficacy among devices were not validated due to the small sample size and relatively short follow-up period. Further long-term, large scaled, randomized, prospective study is warranted to support our conclusion.

## 6. Conclusion

Combined procedure of CBA and LAA closure is a feasible strategy for PAF patients, with comparable safety and efficacy to CBA and OACs. Long-term, largescaled, prospective trials are warranted to provide stronger evidences.

## Figures and Tables

**Table 1:** Baseline characteristics of the patients.

Parameters	CBA only group (*N* = 262)	CBA + LAAC group (*N* = 42)	*p* value
Age (yr)	66.3 ± 9.5	70 ± 7.6	**0.01** ^*∗∗*^
Gender: male	142 (54.2)	26 (61.9)	0.35
Smoking	49 (18.7)	6 (14.3)	0.49
Wine	24 (9.2)	6 (14.3)	0.45
proBNP (pg/ml)	**298.9 (105.4, 776.2)**	**450.8 (85.8, 859.4)**	0.65
eGFR (mL/(min*∗*1.73 m^2^))	81.5 ± 17.1	76.4 ± 16.3	0.07
Left atrial diameter (mm)	40.4 ± 5.6	45.6 ± 5.8	**<0.0001** ^*∗∗*^
LVEF (%)	62.1 ± 8.1	60.9 ± 4.2	0.19
※HAS-BLED score	2.7 ± 1.2	3.7 ± 1.2	**<0.0001** ^*∗∗*^
※CHA_2_DS_2_-VASc score	2.8 ± 1.9	3.8 ± 2.1	**0.001** ^*∗∗*^

*Medical history*			
Hypertension	154 (58.8)	26 (61.9)	0.70
Diabetes mellitus	30 (11.5)	8 (19.1)	0.17
Hyperlipidemia	10 (3.8)	6 (14.3)	**0.01** ^*∗∗*^
Hyperthyroidism	10 (3.8)	1 (2.4)	0.99
Coronary heart disease	64 (24.4)	10 (23.8)	0.93
Myocardial infarction	24 (9.2)	3 (7.1)	0.89
Previous PCI	22 (8.4)	6 (14.3)	0.35
Valvular heart disease	5 (1.9)	1 (2.4)	1.00
Cardiomyopathy	4 (1.6)	2 (4.8)	0.42
◇Previous stroke	62 (23.7)	26 (61.9)	**<0.0001 ** ^*∗∗*^

*Medication*			
Antiarrythmic drugs	79 (30.2)	17 (40.5)	0.14
Anticoagulants	44 (16.8)	12 (28.6)	0.07
Warfarin	32 (12.3)	5 (12.2)	0.99
Antiplatelet agents	69 (27.1)	19 (46.3)	**0.01** ^*∗∗*^
Aspirin	61 (23.3)	17 (40.5)	**0.02** ^*∗∗*^
Statins	41 (15.6)	13 (31.7)	**0.01** ^*∗∗*^
ACEI/ARB	87 (33.2)	17 (40.5)	0.36
CCB	69 (26.3)	14 (33.3)	0.34
*β*-blocker	71 (27.2)	14 (33.3)	0.41

Continuous variables are described as mean ± SD or median (interquantile range) while categorical variables as percentage (%). CBA, cryoablation; LAAC, left atrial appendage closure; LVEF, left ventricle ejection fraction; PCI, percutaneous coronary intervention; ACEI/ARB, angiotensin-converting enzyme inhibitor/angiotensin receptor blocker; CCB, calcium channel blocker. eGFR estimated glomerular filtration rate, calculated by CKD-EPI formula. **※**CHA_2_DS_2_-VASc score is the risk estimation system of stroke in patients with atrial fibrillation, while HAS-BLED score estimates risk of hemorrhage. **◇**Previous stroke encompasses documented cerebral infarction including lacunar infarction or intracerebral hemorrhage with either CT or MRI evidence.

**Table 2:** Cryoablation procedure details.

Parameter	CBA only group (*N* = 262)	CBA + LAAC group (*N* = 42)	*p* value
Procedure time (s)	1061.8 ± 462.2	974.5 ± 304.3	0.47
Nadir (°C)	−46.4 ± 5.8	−43.4 ± 8.6	0.21

Times of freeze per vein (*n*)			
LSPV	1.8 ± 1.1	2.4 ± 2.0	0.28
LIPV	1.6 ± 0.8	1.6 ± 0.6	0.83
RSPV	1.3 ± 0.6	1.1 ± 0.5	0.15
RIPV	1.5 ± 0.9	1.3 ± 0.6	0.37
◆Anatomic variations	13 (5.0)	5 (11.9)	0.16
RMPV	9 (3.4)	3 (7.1)	0.47
◇Ablation of other sites	5(1.9)	0(0)	0.80

Periprocedural complications			
Vagal reflex	29 (11.1)	9 (17.3)	0.20
Phrenic nerve injury	3 (1.2)	0 (0)	1.00
Cardiac tamponade	0 (0)	0 (0)	—

Continuous variables are presented as mean ± SD while categorical variables as percentage (%). PVI, pulmonary isolation; LSPV, left superior pulmonary vein; LIPV, left inferior pulmonary vein; RSPV, right superior pulmonary vein; RIPV, right inferior pulmonary vein; RMPV, right middle pulmonary vein. ◆Anatomic variations include right middle pulmonary vein, left middle pulmonary vein, and absence of inferior pulmonary vein. ◇Other sites ablated include left atrial appendage, superior vena cava, mitral isthmus, and tricuspid isthmus.

**Table 3:** Left atrial appendage occlusion procedure details.

Parameters	CBA + LAAC group (*N* = 42)
*LAA characteristics*	
LAA lobulation, *n* (%)	
One lobe	12 (28.6)
Two lobes	18 (42.9)
Three lobes	3 (7.1)
Multi-lobes	9 (21.4)
LAA opening diameter (mm)	21.2 ± 3.3
LAA depth (mm)	25.3 ± 4.3
*Device characteristics*	
Device company, *n* (%)	
Lefort	22 (52.4)
Lacbes	11 (26.2)
WATCHMAN	9 (21.4)
Device size (mm)	25.0 ± 3.0
Compression ratio (%)	15.9 ± 4.6
*Periprocedural details*	
Implantation, success	41 (97.6)
Released time (s), *n* (%)	
Once	40 (95.2)
Twice	2 (4.8)
Residual flow, *n* (%)	
≤3 mm	5 (12.0)
>3 mm	0 (0)
Cardiac tamponade	0 (0)
◆Electroversion	2 (4.8)

Continuous variables are described as mean ± SD, while categorical variables as percentage (%). CBA, cryoballoon ablation; LAAC, left atrial appendage closure. ◆Electroversion was applied when atrial fibrillation recorded during the procedure.

**Table 4:** Follow-up details.

Parameter	CBA only group (*N* = 262)	CBA + LAAC group (*N* = 42)	*p* value
*Recurrence of AF, n (%)*			
Early recurrence	16 (6.2)	5 (12.0)	0.30
Late recurrence	40 (15.3)	12 (29.3)	**◆0.04** ^*∗∗*^
*LAAC device*			
TEE evaluation, *n* (%)		35 (83.3)	
Successful occlusion		**35 (100)**	
Device-related thrombosis		0 (0)	
Residual flow		0 (0)	
Pericardial effusion		0 (0)	
*Complications, n (%)*			
Death of any cause	2 (0.7)	0(0)	
Rehospitalizaiton due to cardiovascular events	19 (7.3)	2 (4.8)	0.79
Redo-ablation	6 (2.3)	1 (2.4)	1.00
Stroke	2 (0.7)	0 (0)	1.00
Myocardial infarction	2 (0.7)	0 (0)	1.00
Heart failure	0 (0)	0 (0)	—

Categorical variables are described as percentage (%). CBA, cryoballoon ablation; LAAC, left atrial appendage closure; AF, atrial fibrillation; TEE, transesophageal echocardiography. ◆*p* value of late recurrence was generated by the log-rank test.

## Data Availability

The data used to support the findings of this study are available from the corresponding author upon request.

## Supplementary Materials

Supplementary Materials Supplementary file 1: the flow chart of selection and follow-up details.
