# Supplementary figures and images for: Cryoablation Combined with Left Atrial Appendage Closure: A Safe and Effective Procedure for Paroxysmal Atrial Fibrillation Patients
Source: Cardiol Res Pract. 2020 Apr 10;2020:6573296. doi: 10.1155/2020/6573296 (PMC7171640; doi:10.1155/2020/6573296)

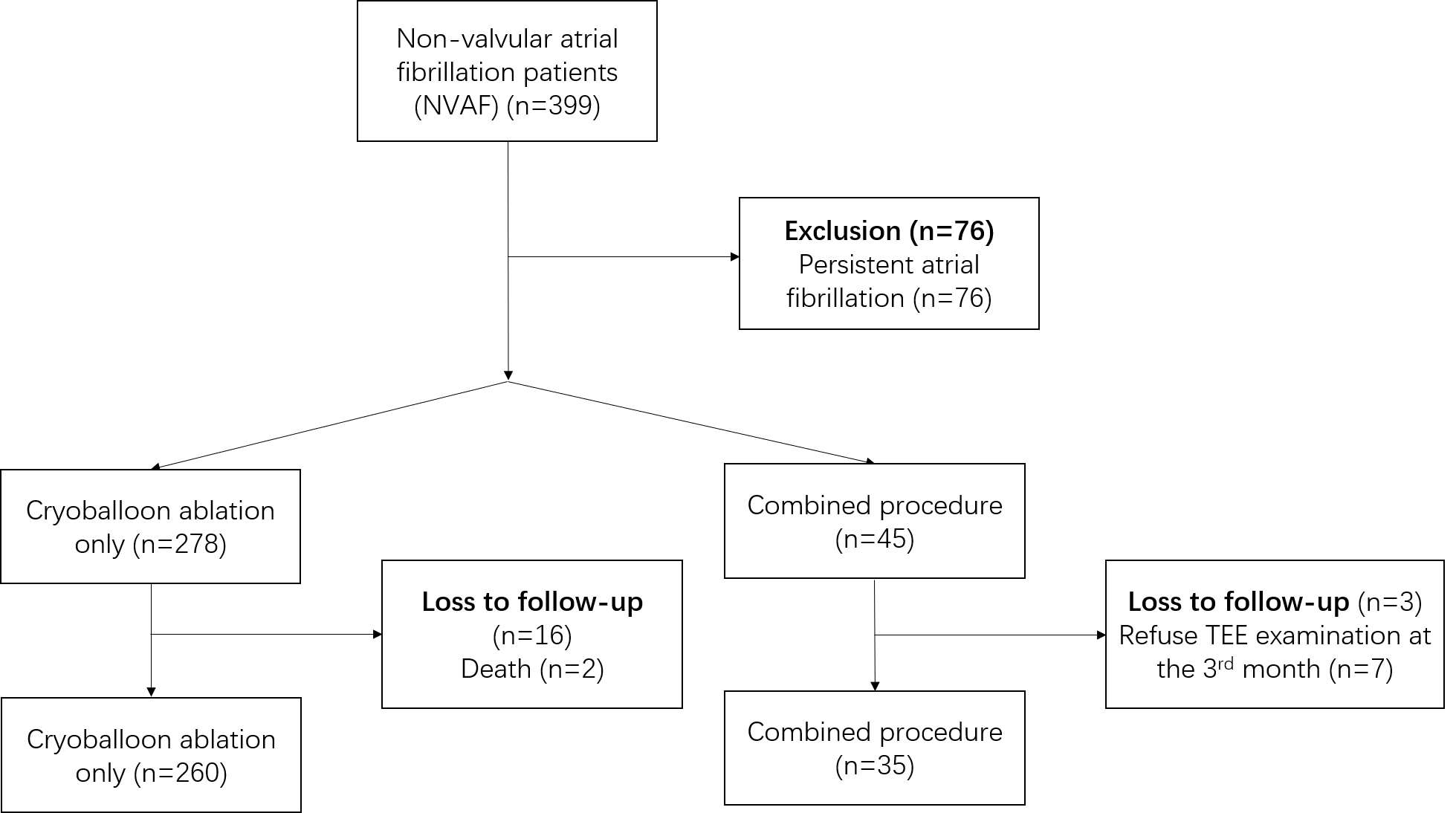

Supplement: Supplementary Materials — Supplementary file 1: the flow chart of selection and follow-up details. [file 6573296.f1.png]
